# The CA125 level postoperative change rule and its prognostic significance in patients with resectable pancreatic cancer

**DOI:** 10.1186/s12885-023-11346-8

**Published:** 2023-09-06

**Authors:** Xin Luo, Xianchao Lin, Ronggui Lin, Yuanyuan Yang, Congfei Wang, Haizong Fang, Heguang Huang, Fengchun Lu

**Affiliations:** Department of General Surgery, Fujian Medical University Union Hospital, 29 Xinquan Road, Fuzhou, 350001 Fujian China

**Keywords:** Resectable pancreatic cancer, CA125, Early recurrence

## Abstract

**Background:**

The relationship between postoperative CA125 level changes and early recurrence after curative resection of resectable PDAC is still unclear.

**Methods:**

The electronic medical records and follow-up data of patients with resectable pancreatic cancer were evaluated. Dynamic CA125 detection was used to identify the rules for postoperative CA125 level change and its prognostic value in patients with resectable pancreatic cancer.

**Results:**

The study included a total of 118 patients with resectable pancreatic cancer who underwent curative resection. Early postoperative CA125 levels were significantly higher than those before surgery (P < 0.05). It decreased gradually in the group without early recurrence (P < 0.05) but not in the early recurrence group (P>0.05). There was no correlation between early postoperative CA125 levels and early recurrence (P > 0.05). CA125 levels three months after surgery were associated with an increased risk of early recurrence (P = 0.038, 95% CI (1.001–1.025)). The cutoff CA125 level at 3 months after surgery for predicting early recurrence was 22.035. Patients with CA125 levels < 22.035 three months postoperatively had similar DFS and OS, regardless of whether the value was exceeded in the early postoperative period, but these values were significantly better than those of patients with CA125 levels > 22.035 at 3 months postoperatively (p < 0.05).

**Conclusions:**

Patients with different prognoses have different patterns of CA125 level changes. Elevations in CA125 levels > 3 months postoperatively, rather than early postoperative elevation, were associated with a poor prognosis.

**Supplementary Information:**

The online version contains supplementary material available at 10.1186/s12885-023-11346-8.

## Introduction

Pancreatic ductal adenocarcinoma (PDAC) is an extremely aggressive malignancy that is the fourth leading cause of cancer-related deaths [1]. Surgical resection remains the only accepted curative treatment for pancreatic ductal adenocarcinoma (PDAC) [2]. Unfortunately, pancreatic cancer has a resectable rate of less than 20% [3]. Moreover, 80% of patients experience disease recurrence after surgery [4]. In fact, one-third of patients experience early recurrence (local recurrence or distant metastasis within 6 months after surgery). The reported median survival time for these patients is only 8.4 to 10.6 months [5, 6].

Several studies have identified the following risk factors for early recurrence (ER) after surgery in patients with PDAC: CRP > 3.0 mg/dL, decrease in total lymphocyte count by > 50% of baseline value, modified Glasgow prognostic score = 2, preoperative CA19-9 > 300 U/ml, tumor size > 30 mm, retroperitoneal invasion, and diabetes mellitus [7–9]. However, the clinical significance of cancer antigen 125 (CA125) for the early recurrence of resectable PDAC remains unclear.

CA125/mucin 16 (MUC16) is a transmembrane mucin. CA125 was initially identified by the monoclonal antibody OC125, which was detected in mice immunized with an ovarian cancer cell line [10], and MUC16 was developed by molecular cloning of CA125 [11]. Since then, CA125/MUC16 has become the most significant biomarker for ovarian cancer diagnosis, surveillance of disease progression, and recurrence [12, 13]. Meanwhile, recent studies have found that pancreatic cancer exhibits an overexpression of CA125 (MUC16) [14]. Overexpression of CA125 can enhance the motility and invasion of PDAC cells [15] and promote liver metastasis [16]. These findings suggest that serum CA125 has clinical utility in monitoring the recurrence and progression of pancreatic cancer. At present, several retrospective studies have reported that preoperative CA125 is associated with the presence of occult metastasis before surgery and can be used to predict early distant metastasis postoperatively in resectable PDAC [17, 18].

However, the relationship between postoperative CA125 levels and early recurrence after resection of resectable PDAC has not been elucidated. In particular, no studies have specifically evaluated the law of dynamic alterations of CA125 following radical resection of pancreatic cancer and its relationship to early recurrence. In this work, we used dynamic CA125 detection to identify the postoperative CA125 level change rule and its prognostic significance in patients with resectable pancreatic cancer.

## Materials and methods

### Patients

This is a retrospective, single-center study of resectable pancreatic cancer patients who underwent curative resection and adjuvant chemotherapy between January 1, 2017, and March 18, 2022. This study included a total of 118 patients, excluding 15 patients who did not receive adjuvant chemotherapy in our center after surgery, 7 patients who did not complete 1 cycle of adjuvant chemotherapy, and 1 patient who died within 30 days. R0 resection was achieved in all patients enrolled in the study. The patient’s electronic medical record and follow-up records were reviewed to obtain the patient’s age, sex, BMI, tumor size, tumor location, preoperative CA199, preoperative CEA, and CA125 levels (including the levels of each test before and after surgery), pathological type, TNM stage, time to begin postoperative chemotherapy, chemotherapy regimen, disease-free survival (DFS), and overall survival (OS). The study protocol was approved by the ethics committee of Fujian Medical University Union Hospital.

### Serum levels of CA 125

Before surgery, CA125 levels were routinely examined. Postoperative testing began before the start of chemotherapy and was performed every two weeks during chemotherapy.

### Treatment

All patients were treated with curative resection, which included pancreaticoduodenectomy and distal pancreatectomy with splenectomy. After surgery, adjuvant chemotherapy (AC) was administered as standard treatment. The modified FOLFIRINOX regimen was as follows: oxaliplatin (85 mg per square meter), irinotecan (150 mg per square meter), leucovorin, (400 mg per square meter), and fluorouracil (2,400 mg per square meter) every 14 days for 24 weeks (12 cycles). AG: nab-paclitaxel (125 mg per square meter) and gemcitabine (1000 mg per square meter) were given on days 1, 8, and 15 every 28 days for 24 weeks.

### Definition

A recent international multicenter study determined that 6 months was the optimal DFS cutoff for distinguishing early recurrence from late recurrence. OS was only 8.4 months (95% CI: 7.3 to 9.6) in the early recurrence (ER) group and 31.1 months (95% CI: 25.7 to 36.4) in the not-early recurrence/no recurrence group [5]. Therefore, this study defined early recurrence as local recurrence or distant metastasis within 6 months after surgery. Not-early recurrence (Not-ER) was defined as recurrence > 6 months or no recurrence. Imaging is utilized to diagnose relapse. The first sites of recurrence to be recorded were divided into six categories: liver-only, lung-only, peritoneum-only, local-only, multiple-sites and other. A local recurrence is defined as a recurrence within the surgical area. Others are defined as less common sites of recurrence. Early period postoperative CA125 is defined as the levels of CA125 before 3 months postoperatively. DFS was defined from the date of surgery to the date of recurrence or the date of the last follow-up without recurrence. OS was defined from the date of surgery to the date of death or the date of the last follow-up. Resectable PDAC was defined as a tumor without arterial (common hepatic artery, celiac axis, and superior mesenteric artery) and superior mesenteric vein (SMV)/portal vein (PV) contact at ≥ 180° or occlusion of the SMV/PV.

### Follow-up

After surgery, the patients were followed up at least every three months for the first year, every three to six months for the second and third years, and every six months thereafter. The follow-up included physical examinations, laboratory tests, tumor markers, computed tomography or magnetic resonance imaging.

### Statistical analysis

Percentages and frequencies were used for the representation of categorical variables. The normality of continuous variables was verified using the Shapiro‒Wilk test. Continuous variables were reported using means and standard deviations (SD) or medians and interquartile ranges (IQR). t tests or the nonparametric Mann‒Whitney U test were used to compare continuous variables based on whether they followed a normal distribution. The chi-square test or Fisher’s exact test was used for the analysis of categorical variables. The multivariable logistic regression analysis with the forward stepwise conditional method was used to determine independent risk factors for ER for variables with a P value < 0.05 by univariate analysis. At the same time, Lasso regression was used to verify the results of univariate logistic regression. A receiver operating characteristic (ROC) curve was constructed to estimate the optimal cutoff value for serum CA125 as a risk factor for early recurrence. The Kaplan–Meier method (log rank test) was used to estimate disease-free survival and overall survival. The data were considered significant at P < 0.05.

## Results

### Patient characteristics

There were a total of 118 patients included in the study, with 41 females (34.7%) and 77 males (65.3%). The majority of the tumors were in the pancreatic head (n = 78, 66.1%) or the tail and body (n = 37, 31.3%), and they had a median size of 3.2 (2.5 to 4.0) cm. After surgery, adjuvant chemotherapy was administered to all patients, 110 (93.2%) of whom were treated with the AG regimen. The duration of median disease-free survival was 11.0 (5.75 to 16.0) months. The duration of median overall survival was 18.5 (14.0 to 26.25). (Table 1).

**Table 1:** Characteristics of 118 patients with resectable pancreatic cancer

Variable	Mean ± SD	N (%)	Median (IQR)
Age(years),			64.0(55.0 to 69.0)
Sex			
Female		41(34.7)	
Male		77(65.3)	
BMI	22.3 ± 3.0		
Tumor seize(cm)			3.2(2.5 to 4.0)
Location			
Head		78(66.1)	
Body and Tail		37(31.3)	
Others		3(2.5)	
Preoperative CA-199			176.7(37.8 to 533.4 )
Preoperative CA-125			14.8(10.8 to 26.9)
Preoperative CEA			3.4(2.1 to 6.0)
Histological type			
Well-mod. adenocarcinoma		103(87.3)	
Poor adenocarcinoma		11(9.3)	
Others		4(3.4)	
TNM stage, n (%)			
IA		5(4.2)	
IB		33(28.0	
IIA		18(15.3)	
IIB		36(30.5)	
III		26(22.0)	
TBCAS(day)			36(30 to 60)
Chemotherapy regimens			
AG		110(93.2)	
mF		8(6.8)	
Recurrence site		73	
Liver-only		39(53.4)	
Lung-only		2(2.7)	
Peritoneum-only		12(16.4)	
Local-only		10(13.7)	
Multiple-site		7(9.6)	
Other		3(4.1)	
DFS(months)			11.0(5.75 to 16.0)
OS(months)			18.5 (14.0 to 26.25)

BMI: Body Mass Index; TBCAS: Time to begin chemotherapy after surgery; DFS: Disease free survival; OS: overall survival

### Chemotherapy adherence

All patients received at least one cycle of chemotherapy. 72% (85/118) of patients received 6 or more cy-cles of chemotherapy. Typically, patients receive six full cycles of chemotherapy. If radiographic metastases are present, pa-tients are advised to continue chemotherapy or change chemotherapy regimens.

### Predictors of early recurrence

The risk factors for early recurrence were screened by univariate logistic regression analysis and LASSO regression analysis (Supplementary Fig. 1). After determining preoperative CA125 as a risk factor for early recurrence in univariate logistics regression, the cut-off value of 26.95 was calculated using ROC curve. Tumor location, pathological vascular invasion, positive lymph node, preoperative CA125 levels and chemotherapy regimen were included in the multivariable analysis. Preoperative CA-125 level>26.95, pathological vascular invasion and positive lymph nodes were found to be independent predictors of early recurrence (Table 2). Patients with pre-operative CA125 levels>26.95 had poorer DFS and similar OS compared to patients with preoperative CA125 levels<26.95(Supplementary Fig. 2).

**Table 2:** Risk factors for early recurrence

Variable	Univariate Analysis, p value	Multivariable Analysis		
	p Value	Odds Ratio	95% CI	p Value
Age(years)	0.987			
Sex	0.458			
Female				
Male				
BMI	0.454			
ALB	0.920			
HB	0.511			
Tumor size(cm)	0.422			
Tumor location (Others VS Head)	**0.012**	1.86	0.65–5.37	0.25
Preoperative CA-199	0.139			
Preoperative CA-125 (>26.95 VS <26.95)	**<0.001**	5.47	1.83–16.4	**0.002**
Preoperative CEA	0.691			
Histological type	0.965			
Well-mod. adenocarcinoma				
Poor adenocarcinoma				
Others				
Pathological vascular invasion (yes vs. no)	**<0.001**	9.27	2.51–34.23	**0.001**
Positive lymph nodes (yes vs. no)	**<0.001**	4.18	1.52–11.5	**0.006**
Chemotherapy regimens (mF vs. AG)	**0.018**	4.2	0.56–31.68	0.164
TBCAS(day)	0.404			

BMI: Body Mass Index; HB: hemoglobin; ALB: albumin; TBCAS: Time to beginning chemotherapy after surgery; TBCAS: Time to begin chemotherapy after surgery

### Postoperative CA125 and early recurrence

**Fig. 1:**
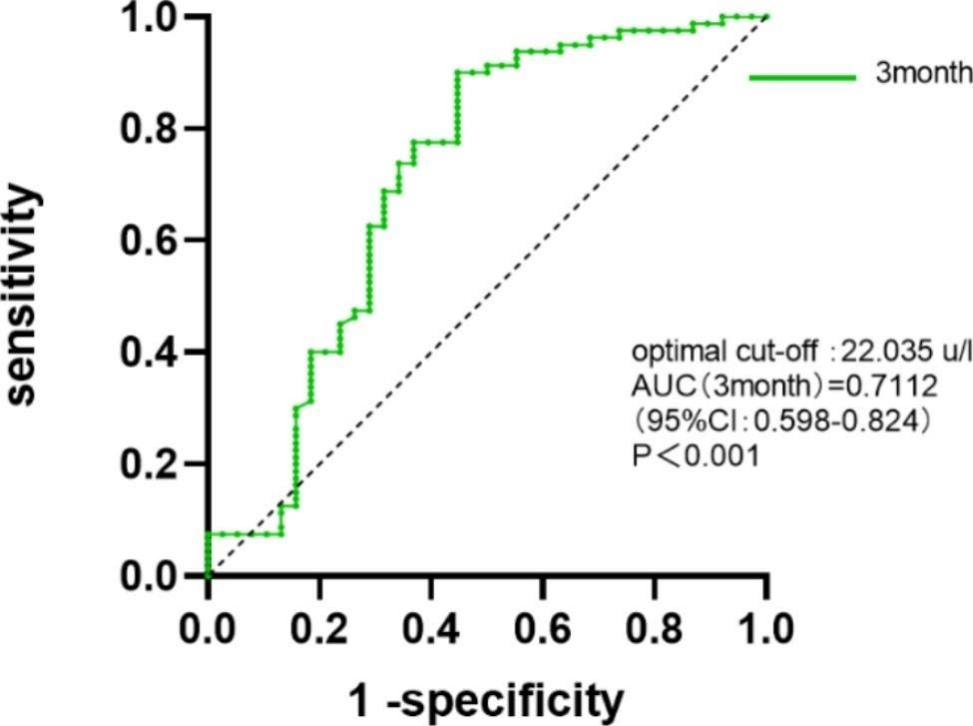
The cutoff CA125 level for predicting early recurrence 3 months after surgery

**Fig. 2:**
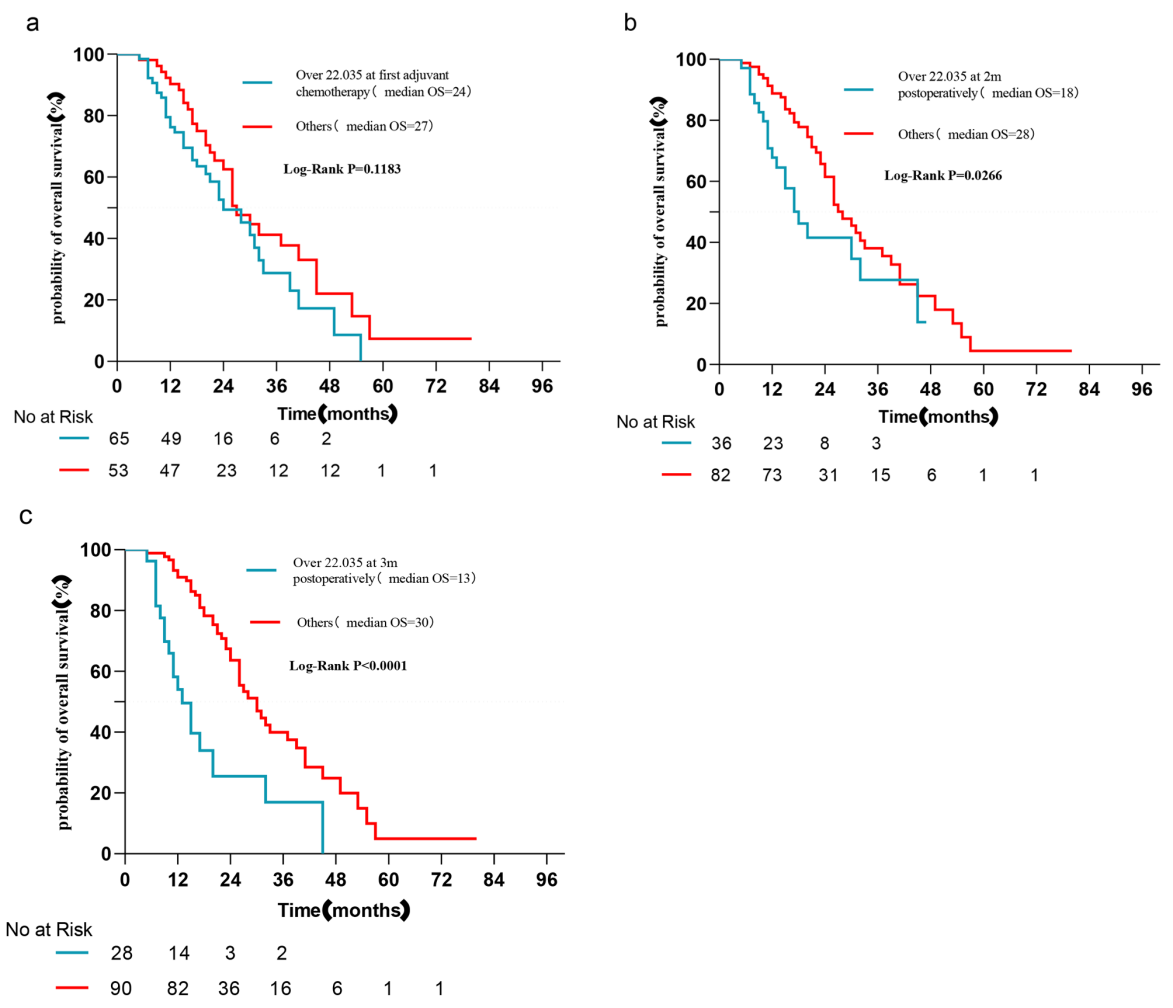
Kaplan‒Meier analyses of the relationship between CA125 > 22.035 at different times and overall survival (a: over 22.035 at first chemotherapy; b: over 22.035 at 2 months postoperatively; C: over 22.035 at 3 months postoperatively). CA125 > 22.035 at the first postoperative chemotherapy was not associated with poor OS. (log rank test)

Dynamic postoperative CA125 detection was performed. CA125 levels at various time points (first adjuvant chemotherapy, two months after surgery, three months after surgery, and four months after surgery) were included in the logistic regression analysis. Three and four months after surgery, an increase in the CA125 level was found to be a risk factor for early recurrence (P < 0.05) (Table 3). Although CA125 levels were elevated in the early postoperative period for the majority of patients compared to the preoperative period (first adjuvant chemotherapy: 89/118; two months after surgery: 60/118), the increase in CA125 levels was not associated with an increased risk of early recurrence (P > 0.05) (Table 3). The area under the ROC curve (AUC) for CA125 3 months after surgery was 0.7112 (95% CI: 0.598–0.824, P < 0.001), and 22.035 U/l was the best threshold for predicting ER, with a specificity of 90.0% and a sensitivity of 55.3% (Fig. 1). Moreover, the proportion of liver metastases in patients with CA125 levels > 22.035 three months after surgery was also significantly higher (60.7% vs. 30.0%, P = 0.003). Similar results were obtained when the Kaplan‒Meier curve was used to analyze the relationship between CA125 > 22.035 and overall survival at different times. CA125 > 22.035 at the first postoperative chemotherapy was not associated with poor OS (P = 0.1183, Fig. 2).

**Table 3:** Postoperative CA125 and early recurrence

CA125 at different time	Univariate Analysis,		
	Odds Ratio	95% CI	p Value
First chemotherapy	1.005	0.998–1.012	0.154
2 months postoperatively	1.005	0.999–1.012	0.109
3 months postoperatively	1.013	1.001–1.025	**0.038**
4 months postoperatively	1.011	1.000-1.023	**0.050**

**Fig. 3:**
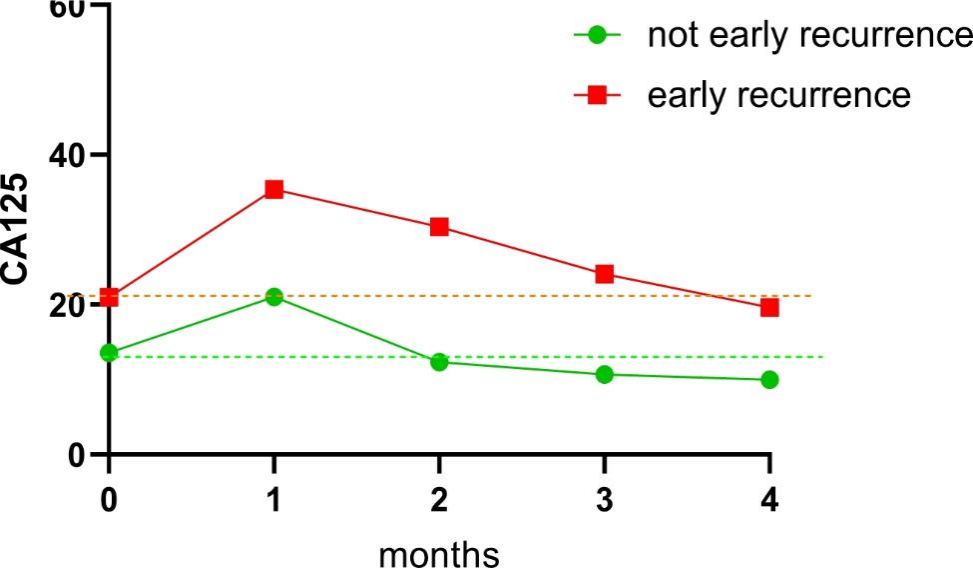
Variation trend of CA125 level. X-axis 0: CA125 preoperatively; X-axis 1: CA125 at the first chemotherapy; X-axis 2: CA125 2 months after surgery; X-axis 3: CA125 3 months after surgery. Early recurrence group: CA125 levels did not decrease significantly after postoperative elevation (P > 0.05) and were still higher than preoperative levels at 3 months after surgery. Not-early recurrence group: CA125 levels decreased gradually (p<0.05) and dropped below the preoperative level two months after surgery

The patients were divided into an early recurrence group and a nonearly recurrence group, and the change regularity of postoperative CA125 was analyzed further. At the beginning of adjuvant chemotherapy, the CA125 levels of both groups were significantly higher than those before surgery (P < 0.05). In the early recurrence group, the CA125 levels did not decrease significantly after postoperative elevation (P > 0.05) and were still higher than the preoperative level at 3 months after surgery (Fig. 3). In contrast, the CA125 levels in the not-early recurrence group decreased gradually (p<0.05). and dropped below the preoperative level two months after surgery (Fig. 3). In contrast, the CA125 levels in the not-early recurrence group decreased gradually (p < 0.05). and dropped below the preopera-tive level two months after surgery (Fig. 3). However, the CEA and CA199 levels of patients after the first postoperative chemotherapy were significantly decreased compared with those of patients before surgery. At four months after surgery, CA199 and CEA levels were relatively stable in the Not-ER group, while the ER group showed a slow upward trend (Supplementary Fig. 5). Postoperative CA125 was significantly higher than that before surgery, but it gradually decreased in the group without early recurrence, while it remained elevated in the group with early recurrence (Table 4).

**Table 4:** Variation trend of CA125 level (Not early recurrence group VS early recurrence group)

	Not early recurrence			Early recurrence		
Group	Median (IQR)	Median (IQR)	P value	Median (IQR)	Median (IQR)	P value
Group A VS Group B	13.59(10.23 to 19.28)	21.04(13.67–37.84)	**<0.001**	20.96(13.56–45.70)	35.36(22.71–66.89)	**P = 0.01**
Group B VS Group C	21.04(13.67–37.84)	12.31(9.56 to 19.92)	**<0.001**	35.36(22.71–66.89)	30.38(12.38–45.19)	0.055
Group C VS Group D	12.31(9.56 to 19.92)	10.69(8.55 to 15.23)	**0.016**	30.38(12.38–45.19)	24.09(9.95–53.33)	P = 0.516

Group A: preoperative CA125. Group B: CA125 of the first chemotherapy. Group C: 2 months after surgery. Group D: 3 months after surgery. Postoperative CA125 is significantly higher than that before surgery, but it will gradually decrease in the group without early recurrence, while the level of CA125 in the group with early recurrence may remain elevated

**Fig. 4:**
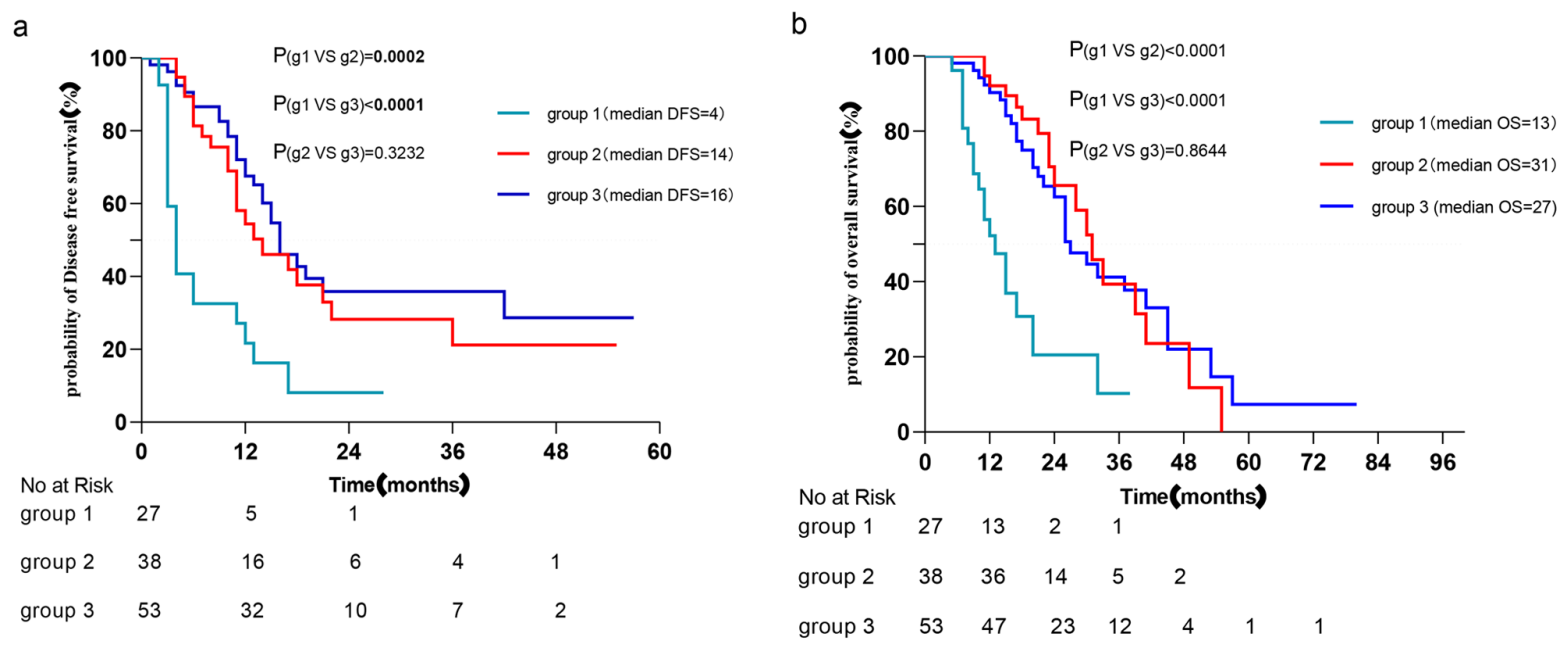
Kaplan‒Meier analyses of the OS and DFS of patients with different variation trends of CA125 postoperatively. Group 1: It was over 22.035 for longer than 3 months postoperatively. Group 2: It was over 22.035 in the early postoperative period and decreased to below 22.035 within three months. Group 3: It remained below 22.035 3 months postoperatively. (log rank test). Patients with CA125 levels < 22.035 at three months postoperatively had similar DFS and OS, regardless of whether the value was exceeded in the early postoperative period but were significantly better than patients with CA125 levels > 22.035 at 3 months postoperatively

The groupings were according to the variation in CA125 level: Group 1: CA125 was over 22.035 for longer than 3 months postoperatively. Group 2: It was over 22.035 in the early postoperative period and decreased to below 22.035 within three months. Group 3: It remained below 22.035 3 months postoperatively. The DFS and OS of the three groups were compared in pairs through the Kaplan‒Meier curve. The DFS and OS of Group 2 and Group 3 were better than those of Group 1 (P < 0.05), but there was no significant difference between Group 2 and Group 3. The increase in CA125 in the early postoperative period is not directly related to a poor prognosis. If it can be reduced to below 22.035 three months after surgery, regardless of whether it is greater than 22.035 in the early postoperative period, then the patient still has a relatively good prognosis (Fig. 4).

### The time to recurrence found on imaging vs. the time of elevation of CA125

A total of 73 patients with imaging recurrence were included. The time when CA125 first exceeded 22.035 within 1 year after surgery, and the corresponding time of recurrence on imaging was compared (if it was still greater than 22.035 three months after surgery, the time of first elevation was calculated; if it fell below 22.035 within 3 months, the time of second ele-vation was calculated). The time point of CA125 elevation within 1 year (49/72) after surgery was earlier than the time of recurrence as confirmed by imaging, which was statistically significant (P < 0.05) (Table 5, Supplementary Fig. 4).

**Table 5:** The time when CA125 was first > 22.035 within 1 year postoperatively vs. the time when recurrence was detected on imaging

Time of first elevation of CA125	CA125 (month), median (IQR))	imaging(month), median (IQR))	P value
Within 1 months	1(1 to 1)	4(3 to 6)	**<0.001**
Within 2 months	1(1 to 1.75)	4(3 to 6)	**<0.001**
Within 3 months	1(1 to 2)	4(3 to 6)	**<0.001**
Within 4 months	1(1 to 2)	4(3 to 6)	**<0.001**
Within 5 months	1(1 to 2.5)	4(3 to 6)	**<0.001**
Within 6 months	2(1 to 6)	4(3 to 11.5)	**0.002**
Within 7 months	2.5(1 to 6)	4(3 to 11.0)	**0.005**
Within 8 months	3.5(1 to 6)	5(3 to 10.5)	**0.012**
Within 9 months	5(1 to7)	5.5(3 to 11)	**0.017**
Within 10 months	6(1 to 8)	6(3 to 11)	**0.019**
Within 11 months	6(1 to 8)	6(3 to 11)	**0.029**
Within 12 months	6(1.75 to 9)	6(3.75 to 11.25)	**0.033**

## Discussion

In this study, we detected the CA125 level postoperative change rule and its prognostic significance in patients with resectable pancreatic cancer. Our study found that CA125 levels were elevated in the early postoperative period for the majority of patients compared to the preoperative period. However, there was no correlation between early postoperative CA125 levels and early recurrence or poor OS (P > 0.05). Three months after surgery, elevated CA125 levels were a risk factor for early recurrence. The level of CA125 was not persistently elevated in the not-early recurrence group. It increased in the early postoperative period and then decreased. As long as the value falls below 22.035 within 3 months after surgery, a relatively good prognosis can be obtained regardless of whether the value is exceeded in the early postoperative period. In addition, the initial elevation of CA125 within 12 months after surgery significantly precedes the time of recurrence as detected by imaging.

Less research has been conducted on the diagnostic and prognostic value of CA125 in pancreatic cancer. Previous studies have confirmed that high baseline CA125 levels predict early distant metastasis after pancreatectomy and are associated with the presence of occult metastasis before surgery [17, 19]. Our study shows that preoperative CA125 > 26.95 is associated with early recurrence and poor DFS. Previous studies have also confirmed that postoperative elevation of CA125 is a risk factor for early recurrence, particularly liver recurrence [17, 20]. These studies, however, only analyzed CA125 levels at a single timepoint. In clinical diagnosis and treatment, we usually conduct dynamic detection of tumor markers in patients, so the study of the dynamic change pattern of CA125 after pancreatic cancer surgery may have higher clinical significance.

The level of CA125 in the initial postoperative period of pancreatic cancer was significantly higher than that before surgery. However, our data confirmed that the increase in CA125 levels within 3 months after surgery was not directly related to early recurrence and that the increase in CA125 levels within 2 months after surgery was not directly related to poor OS. To our knowledge, we are the first study to report these results. Exactly what causes this phenomenon has not been confirmed. At present, the mechanism of CA125 secretion is not fully understood. One study proposed that the release or secretion of CA125 is directly related to the epithelial growth factor receptor (EGFR) signal transduction pathway [21]. Prior studies have shown that EGFR plays a crucial role in cell proliferation and wound healing [22–24]. More importantly, recent studies have confirmed that EGFR activation promotes pancreatic healing in patients with pancreatitis [25]. Therefore, we cautiously suggest that the early postoperative elevation of CA125 is partly due to the activation of epithelial growth factor receptors by the need for postoperative cell repair and proliferation. This may explain why the level of CA125 is generally increased in the early postoperative period. At the same time, we observed that patients with unresectable pancreatic cancer also showed a transient elevation of CA125 after laparoscopic pancreatic tumor biopsy. However, no similar findings were found after surgery for benign pancreatic tumors. Previous studies have found that CA125/MUC16 is not expressed in normal pancreas but is upregulated in primary and metastatic pancreatic cancer tumors [14]. Therefore, we cautiously believe that the high expression of CA125/MUC16 is also one of the conditions for this phenomenon. Regarding the mechanism of this interesting phenomenon, we will perform further research in the future.

However, early postoperative CA125 levels are not associated with early recurrence of pancreatic cancer. Therefore, to de-termine when they reflect the risk of tumor recurrence, we dynamically monitored CA125 levels to determine the relationship between their pattern of change and early recurrence.

After a transient increase postoperatively, CA125 levels in patients without early recurrence exhibited a significant downward trend, falling below the preoperative level by the second month after surgery. The CA125 levels of patients in the early recurrence group did not decrease significantly after the postoperative increase and remained higher than the preoperative level three months after surgery. Therefore, we believed that the influence of EGFR signaling pathway activation on CA125 levels would diminish over time following the completion of patients’ postoperative repair. Then, after the effect disappeared, CA125 levels decreased rapidly in patients with a low risk of recurrence. Consequently, the CA125 level after a certain time postoperatively can more accurately reflect the risk of tumor recurrence and metastasis. As long as the CA125 level falls below 22.035 U/L three months after surgery, regardless of whether this value was exceeded in the early postoperative period, the DFS and OS of these patients were significantly better than those of patients whose CA125 levels remained above 22.035 U/L for more than three months after surgery. Considering the above discussion that the CA125 level three months after surgery is a risk factor for early recurrence, we believe that the interference of other factors on the CA125 level gradually disappears at approximately three months after surgery. The predictive value of CA125 levels after 3 months was higher for postoperative recurrence of pancreatic cancer. Therefore, we suggest that for patients with elevated CA125 in the early postoperative period, there is no direct correlation with a poor prognosis, but the detection density of CA125 should be increased, and if the CA125 level is still greater than 22.035 approximately 3 months after surgery, the patient should undergo further evaluation. At this time, we should be careful regarding the occurrence of early recurrence, and imaging examinations can be actively performed to make a definitive diagnosis and provide patients with better treatments.

Previous studies have found that CA125/MUC16 is not expressed in normal pancreas but is upregulated in primary and metastatic pancreatic cancer tumors [14]. By binding with mesenchymal cells, CA125/MUC16 can enhance the invasion and motility of pancreatic cancer cells [15], and knockout of MUC16 can reduce the growth and metastasis of pancreatic cancer [26]. Recent studies have also found that MUC16 promotes the occurrence of hepatic metastasis of pancreatic ductal adenocarcinoma [16]. These studies indicate that the increase in CA125 is the “cause” and that the metastasis and recurrence of pancreatic cancer is the “effect”. From the above studies, it can be inferred that the increase in CA125 levels should precede the occurrence of recurrence. Our results, in which the elevation of CA125 usually occurs before positive results are found on imaging, are the first results to confirm this hypothesis. Moreover, the proportion of liver metastases in patients with CA125 levels > 22.035 three months after surgery was also significantly higher (60.7% vs. 30.0%, P = 0.003). However, this may be partly because postoperative imaging tests are far less frequent than tumor markers. However, from the perspective of causation, the early elevation of CA125 suggests that we should be highly vigilant about early recurrence, and imaging should be actively performed. In short, CA125 can indeed be used to predict postoperative recurrence of pancreatic cancer, especially liver metastasis.

The limitations of single-center retrospective studies are unavoidable. The cutoff CA125 level for predicting early recurrence of PDAC from single-center data may not be universally applicable. However, the postoperative CA125 change pattern is more clinically significant. We are unable to determine whether the pattern of change in CA125 is related to treatment regimens because only a small proportion of patients are treated with the mF regimen.

## Conclusions

Patients with different prognoses have different patterns of CA125 level changes. A duration of elevation of CA125 levels > 3 months postoperatively, rather than early postoperative elevation, was associated with a poor prognosis.

### Electronic supplementary material

Below is the link to the electronic supplementary material.

Supplementary Material 1

Supplementary Material 2

## Data Availability

The datasets used and analysed during the current study available from the corresponding author on reasonable request.

## Abbreviations

ER: Early recurrence
PDAC: Pancreatic ductal adenocarcinoma
AC125: Cancer antigen 125
AC: Adjuvant chemotherapy
TBCAS: Time to begin chemotherapy after surgery
DFS: Disease free survival
OS: Overall survival
SMV: Superior mesenteric vein
PV: Portal vein
ROC: Receiver operating characteristic

## References

[1] Sung H (2021). Global Cancer Statistics 2020: GLOBOCAN estimates of incidence and Mortality Worldwide for 36 cancers in 185 countries. CA Cancer J Clin.

[2] Neoptolemos JP (2001). Influence of resection margins on survival for patients with pancreatic cancer treated by adjuvant chemoradiation and/or chemotherapy in the ESPAC-1 randomized controlled trial. Ann Surg.

[3] Siegel RL, Miller KD, Jemal A (2020). Cancer statistics, 2020. CA Cancer J Clin.

[4] Groot VP (2018). Patterns, timing, and predictors of recurrence following pancreatectomy for pancreatic ductal adenocarcinoma. Ann Surg.

[5] Seelen LWF (2022). Early Recurrence after Resection of locally Advanced Pancreatic Cancer following induction therapy: an International Multicenter Study. Ann Surg.

[6] Matsumoto I (2015). Postoperative serum albumin level is a marker of incomplete adjuvant chemotherapy in patients with pancreatic ductal adenocarcinoma. Ann Surg Oncol.

[7] Ono S (2022). Predictive factors for early recurrence after pancreaticoduodenectomy in patients with resectable pancreatic head cancer: a multicenter retrospective study. Surgery.

[8] Matsumoto I (2015). Proposed preoperative risk factors for early recurrence in patients with resectable pancreatic ductal adenocarcinoma after surgical resection: a multi-center retrospective study. Pancreatology.

[9] Kim NH, Kim HJ (2018). Preoperative risk factors for early recurrence in patients with resectable pancreatic ductal adenocarcinoma after curative intent surgical resection. Hepatobiliary Pancreat Dis Int.

[10] Bast RC (1981). Reactivity of a monoclonal antibody with human ovarian carcinoma. J Clin Invest.

[11] Yin BW, Lloyd KO (2001). Molecular cloning of the CA125 ovarian cancer antigen: identification as a new mucin, MUC16. J Biol Chem.

[12] Felder M (2014). MUC16 (CA125): tumor biomarker to cancer therapy, a work in progress. Mol Cancer.

[13] Bocheva Y, Bochev P, Ivanov S (2015). Ca-125 in diagnosis and monitoring of patients with ovarian cancer. Akush Ginekol (Sofiia).

[14] Haridas D et al. *Pathobiological implications of MUC16 expression in pancreatic cancer* PLoS One, 2011. 6(10): p. e10.1371/journal.pone.0026839. 10.1371/journal.pone.0026839 PMC3204976 22066010

[15] Chen SH (2013). Mesothelin binding to CA125/MUC16 promotes pancreatic cancer cell motility and invasion via MMP-7 activation. Sci Rep.

[16] Marimuthu S (2022). MUC16 promotes Liver Metastasis of Pancreatic Ductal Adenocarcinoma by Upregulating NRP2-Associated Cell Adhesion. Mol Cancer Res.

[17] Liu L (2016). Serum CA125 is a novel predictive marker for pancreatic cancer metastasis and correlates with the metastasis-associated burden. Oncotarget.

[18] Cheng H (2021). Predictive values of preoperative markers for Resectable pancreatic body and tail Cancer determined by MDCT to Detect Occult Metastases. World J Surg.

[19] Liu W (2021). Predicting early recurrence for resected pancreatic ductal adenocarcinoma: a multicenter retrospective study in China. Am J Cancer Res.

[20] Tong J (2022). Development and validation of a nomogram to predict liver metastasis for pancreatic ductal adenocarcinoma after radical resection. Front Oncol.

[21] O’Brien TJ (1998). More than 15 years of CA 125: what is known about the antigen, its structure and its function. Int J Biol Markers.

[22] Schlessinger J. Receptor tyrosine kinases: legacy of the first two decades. Cold Spring Harb Perspect Biol. 2014;6(3). 10.1101/cshperspect.a008912. 10.1101/cshperspect.a008912 PMC3949355 24591517

[23] Lemmon MA, Schlessinger J (2010). Cell signaling by receptor tyrosine kinases. Cell.

[24] Hult EM (2022). Myeloid- and epithelial-derived heparin-binding epidermal growth factor-like growth factor promotes pulmonary fibrosis. Am J Respir Cell Mol Biol.

[25] Wen HJ (2019). Myeloid cell-derived HB-EGF drives tissue recovery after pancreatitis. Cell Mol Gastroenterol Hepatol.

[26] Muniyan S (2016). MUC16 contributes to the metastasis of pancreatic ductal adenocarcinoma through focal adhesion mediated signaling mechanism. Genes Cancer.

