# Supplementary material for: The CA125 level postoperative change rule and its prognostic significance in patients with resectable pancreatic cancer
Source: BMC Cancer. 2023 Sep 6;23:832. doi: 10.1186/s12885-023-11346-8 (PMC10481615; doi:10.1186/s12885-023-11346-8)
Supplement: Supplementary file 1 — Supplementary Material 1 [file 12885_2023_11346_MOESM1_ESM.docx]

**Supplementary Table1 ：Baseline characteristics of not early recurrence and early recurrence**

| **Variable** | **Not ER group （n=80）** | **ER group (n=38)** | **p** |
| --- | --- | --- | --- |
| **Age** | 64 (55,69) | 63 (57,68.75) | 0.791 |
| **BMI** | 22.2±2.99 | 22.41±2.92 | 0.717 |
| **Location：** |  |  | **0.026** |
| **Head** | 59 (73.75%) | 19 (50%) |  |
| **Body and Tail** | 19 (23.75%) | 18 (47.37%) |  |
| **Others** | 2 (2.5%) | 1 (2.63%) |  |
| **Tumor seize(cm)** | 3.15 (2.5,4) | 3.5 (2.6,4) | 0.308 |
| **CA199** | 143.8 (35.34,387.8) | 309.4 (69.35,727.15) | 0.080 |
| **CA125** | 13.55 (10.05,18.7) | 20.96 (13.74,43.87) | **0.001** |
| **CEA** | 3.4 (2.08,5.85) | 3.4 (2.15,5.82) | 0.938 |
| **Histological type** |  |  | 1.000 |
| **Well-mod. adenocarcinoma** | 70 (87.5%) | 34 (89.47%) |  |
| **Poor adenocarcinoma** | 8 (10%) | 3 (7.89%) |  |
| **Others** | 2 (2.5%) | 1 (2.63%) |  |
| **TNM stage，n（%）** |  |  | 0.872 |
| **IA** | 5 (6.25%) | 1 (2.63%) |  |
| **IB** | 23 (28.75%) | 9 (23.68%) |  |
| **IIA** | 12 (15%) | 6 (15.79%) |  |
| **IIB** | 23 (28.75%) | 13 (34.21%) |  |
| **III** | 17 (21.25%) | 9 (23.68%) |  |
| **Vascular invasion** |  |  | ＜0.001 |
| **Yes** | 75 (93.75%) | 23 (60.53%) |  |
| **No** | 5 (6.25%) | 15 (39.47%) |  |
| **Positive lymph nodes** |  |  | ＜0.001 |
| **Yes** | 54 (67.5%) | 10 (26.32%) |  |
| **No** | 26 (32.5%) | 28 (73.68%) |  |
| **Chemotherapy regimens** |  |  | **0.022** |
| **AG** | 78 (97.5%) | 32 (84.21%) |  |
| **mF** | 2 (2.5%) | 6 (15.79%) |  |
| **TBCAS，（day）** | 36 (30,49.25) | 34 (27,51) | 0.684 |
| **DFS，（months）** | 14 (11,20.25) | 4 (3,5.75) | **<0.001** |
| **OS，（months）** | 22 (16,32) | 11.5 (8.25,18) | **<0.001** |

BMI：Body Mass Index; TBCAS ：Time to begin chemotherapy after surgery；DFS：Disease free survival；OS：overall survival.
