# Supplementary material for: The CA125 level postoperative change rule and its prognostic significance in patients with resectable pancreatic cancer
Source: BMC Cancer. 2023 Sep 6;23:832. doi: 10.1186/s12885-023-11346-8 (PMC10481615; doi:10.1186/s12885-023-11346-8)
Supplement: Supplementary file 2 — Supplementary Material 2 [file 12885_2023_11346_MOESM2_ESM.docx]

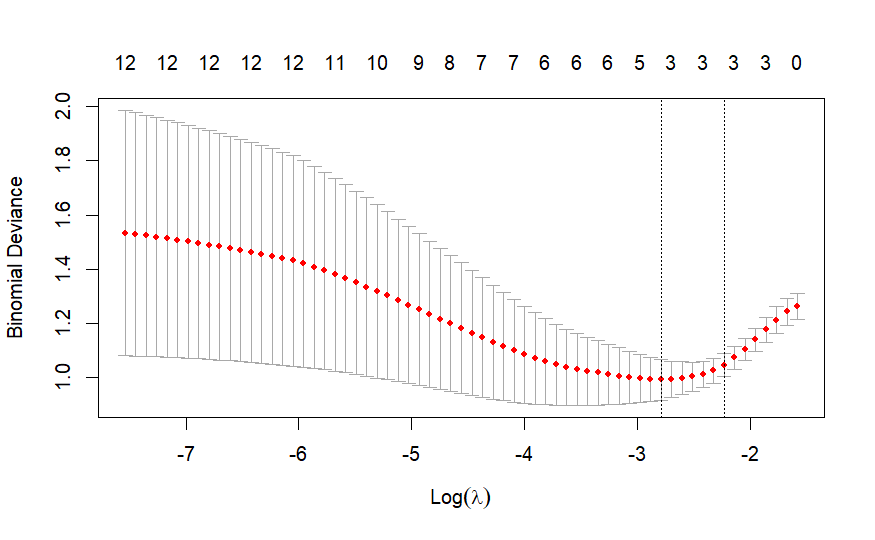


**A**


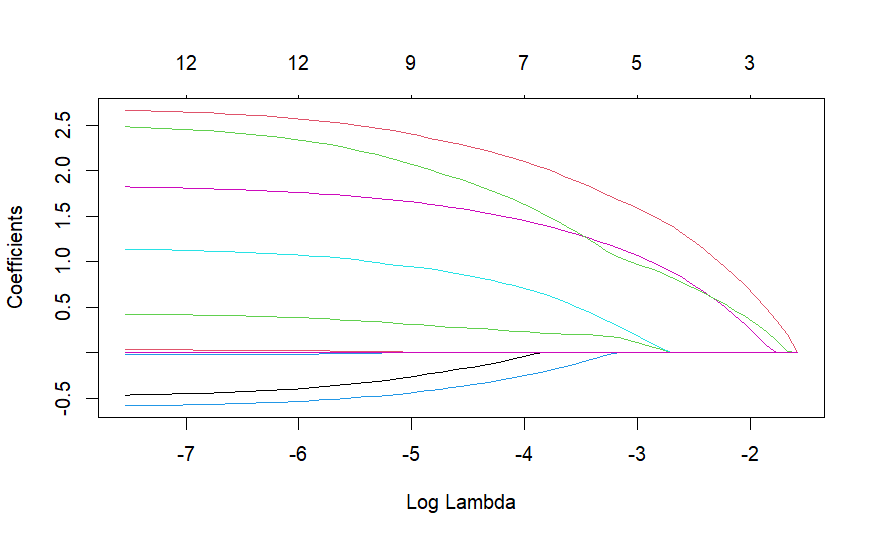


**B**

**Supplementary Figure 1：**The dotted line on the left in panel (A) (lambda.min) refers to the mean value of the smallest target parameter among all λ values. The dotted line on the right in panel (A) (lambda.1 se) refers to the value λ of the simplest model within a variance range of lambda.min. A λ value of 0.04632059 was chosen according to 10-fold cross-validation, and 5 varia-bles were selected for further logistic regression analysis.


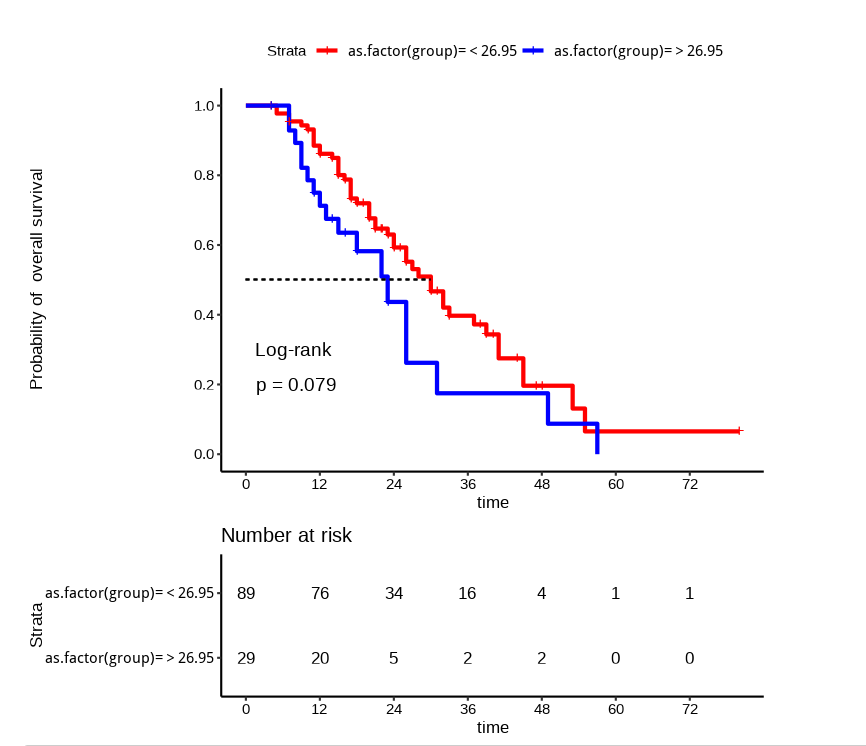

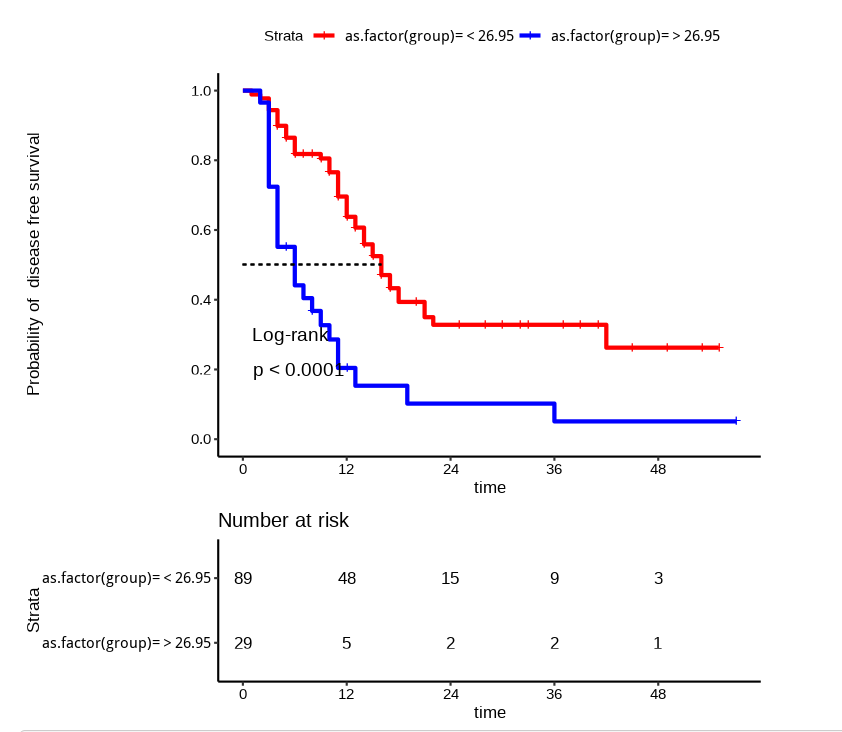


A B

**Supplementary Figure 2.**: Kaplan‒Meier analyses of the OS and DFS of patients with different preoperative CA125 levels. (A) Patients with preoperative CA125 levels >26.95 had poorer DFS and similar OS compared to patients with preoperative CA125 levels <26.95.


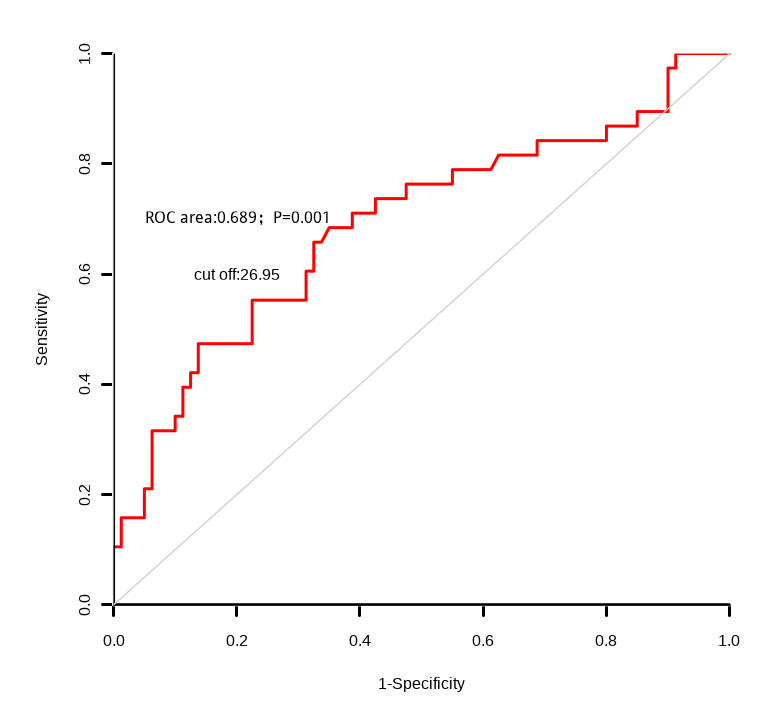


**Supplementary Figure 3**: The cutoff preoperative CA125 level for predicting early recurrence.


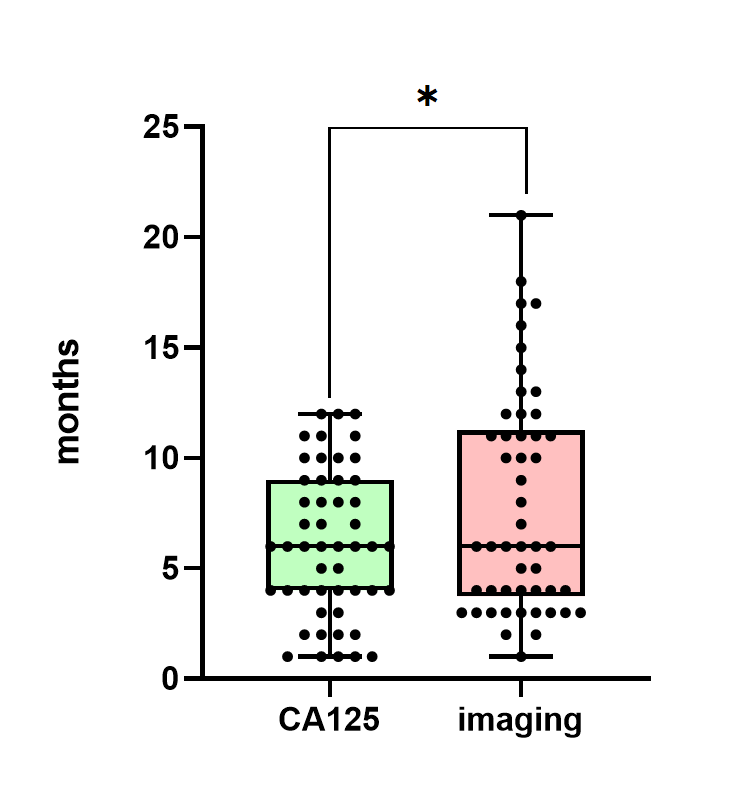


**Supplementary Figure 4:** CA125 was first > 22.035 within 1 year after surgery compared with the time when metastases were detected on its corresponding radiographs. The elevation of CA125 preceded positive imaging results (P<0.05).


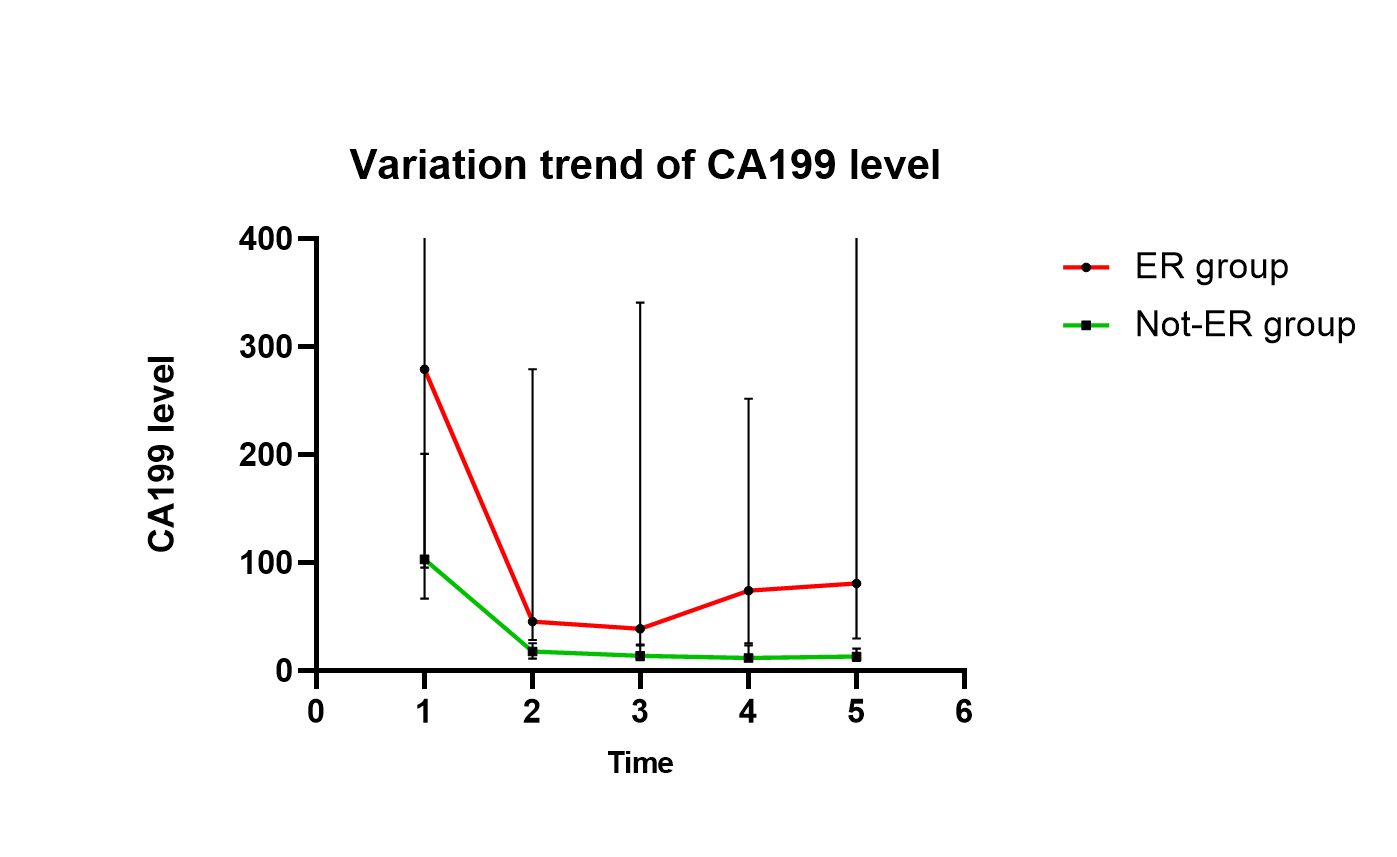


A


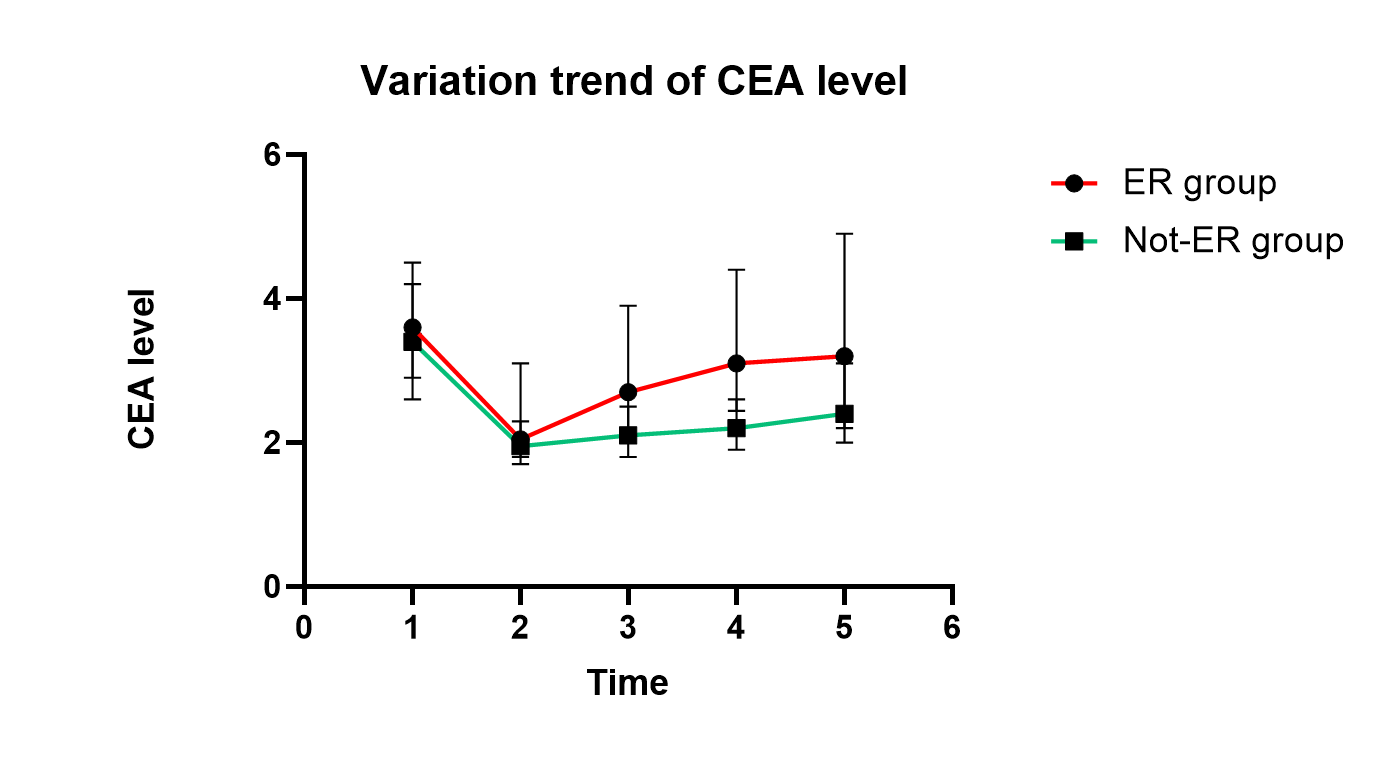


B

**Supplementary Figure 5:** Variation trend of CA199 and CEA levels. X-axis 0: preoperative; X-axis 1: the first chemotherapy; X-axis 2: 2 months after surgery; X-axis 3: 3 months after surgery. At four months after surgery, CA199 and CEA levels were relatively stable in the Not-ER group, while the ER group showed a slow upward trend.
